# High short-term and long-term excess mortality in geriatric patients after hip fracture: a prospective cohort study in Taiwan

**DOI:** 10.1186/1471-2474-15-151

**Published:** 2014-05-09

**Authors:** Li-Wei Hung, Wo-Jan Tseng, Guey-Shiun Huang, Jinn Lin

**Affiliations:** 1 Department of Orthopedic Surgery, National Taiwan University Hospital, Taipei, Taiwan, No.7 Chung-Shan S. Rd, Taipei, Taiwan, 100; 2 Department of Orthopedics Surgery, National Taiwan University Hospital Hsin-Chu Branch, Hsinchu, Taiwan, No.25, Lane 442, Sec. 1, Jingguo Rd., Hsinchu, Taiwan; 3 Department of Nursing, National Taiwan University College of Medicine, Taipei, Taiwan, No.1 Jen-Ai Rd, Sec. 1, Taipei, Taiwan, 100

**Keywords:** Hip fracture, Excess mortality, Osteoporosis, Elderly population

## Abstract

**Background:**

Hip fracture has a high mortality rate, but the actual level of long-term excess mortality and its impact on population-wide mortality remains controversial. The present prospective study investigated short- and long-term excess mortality after hip fractures with adjustment of other risk factors. We calculated the population attributable risk proportion (PARP) to assess the impact of each risk factor on excess mortality.

**Methods:**

We recruited 217 elders with hip fractures and 215 age- and sex-matched patients without fractures from the geriatric department of the same hospital. The mean follow-up time was 46.1 months (range: 35 to 57 months). We recorded data on 55 covariates, including baseline details about health, function, and bone mineral density. We used the multivariate Cox proportional hazards model to analyze hazard ratios (HRs) of short-term (<12 months follow-up) and long-term (≧12 months follow-up) excess mortality for each covariate and calculated their PARP.

**Results:**

Patients with hip fractures had a higher short-term mortality than non-fractured patients, and the long-term excess mortality associated with hip fracture remained high. The significant risk factors for short-term mortality were hip fracture, comorbidities, and lower (below cutoff) Mini Mental State Examination score with HRs of 2.4, 2.3, and 2.3, respectively. Their PARPs were 44.7%, 38.1%, and 34.3%, respectively. The significant risk factors for long-term mortality were hip fracture (HR: 2.7; PARP: 48.0%), lower T-score (HR: 3.3; PARP: 36.2%), lower body mass index (HR: 2.5; PARP: 42.8%), comorbidities (HR: 2.1; PARP: 34.8%), difficulty in activities of daily living (HR: 1.9; PARP: 31.8%), and smoking (HR: 2.5; PARP: 19.2%).

**Conclusions:**

After comprehensive adjustment, hip fracture was a significant risk factor and contributed the most to long-term as well as short-term excess mortality. Its adequate prevention and treatment should be targeted.

## Background

Hip fractures are a major cause of mortality in the elderly. Over 80% of such injuries are caused by low-energy trauma in patients with underlying osteoporosis [1,2]. Globally, 1.6 million osteoporotic hip fractures and 740,000 deaths associated with this injury per year have been reported [3]. Although the incidence of hip fractures has been decreasing in some regions, such as North America and Australia, it is still increasing in Asia, Latin America, the Middle East, and some parts of Europe [4,5]. Given the global increase in the elderly population and the rising number of patients with osteoporosis, the social and economic burden caused by hip fractures is likely to become enormous [3,4,6].

Higher mortality immediately after hip fracture is well documented, and almost all studies have reported significant short-term excess mortality after this injury [7-12]. Excess mortality after hip fracture is defined as a death rate that is higher than that in the population without hip fracture. The risk of death decreases with time, but how long exactly the excess mortality persists after hip fracture has not been settled. Some suggest it does not last long [13-16], while others say it may persist for as long as 10 years [12,17,18].

Many studies have investigated the risk factors potentially associated with excess mortality after hip fracture. But the impact of specific risk factors on excess mortality has not been adequately studied. The purpose of our study was three-fold: to investigate short- and long-term excess mortality after hip fractures, to explore the risk factors of excess mortality, and to calculate the population attributable risk proportion (PARP) for each risk factor. This PARP represented the impact of each risk factor on excess mortality of population after hip fracture.

## Methods

### Ethics statement

The human ethical committees of National Taiwan University Hospital (9361700433) and National Health Research Institutes (EC 0930307) approved this study. We obtained written consent from all study participants.

### Study participants

This prospective observational study included patients with and without hip fracture treated at the National Taiwan University Hospital. Included in the group with hip fractures were patients aged 60 years or older who had a hip fracture. Excluded from the study were patients with pathologic fracture, with previous hip fracture, with previous surgery on the fractured hip, whose fracture was not related to low energy trauma, or who were institutionalized. Low-energy trauma is defined as injury resulting from a transfer of energy that is equal to or less than that of a fall from a standing position. Between April 1, 2004, and January 31, 2006, we recruited 376 consecutive patients with hip fractures in our hospital. After exclusion of 76 patients with previous hip fractures or surgeries, 25 patients whose fracture was not from low-energy trauma, and 13 institutionalized patients, there were 262 eligible patients, among whom 217 agreed to join the study. We tracked the cohort through 2008 for mortality outcome.

Two orthopedic surgeons reviewed the plain films of the injured hips, and they made the diagnosis of hip fracture by consensus. Non-displaced or impacted cervical fractures were fixed with multiple pins or compression hip screws. Displaced cervical fractures were treated with hemiarthroplasty. Fractures over the trochanteric region were fixed with compression hip screws or cephaomedullary nails. All patients in the study group were interviewed for demographic and clinical data when their condition was stable, usually within 6 days on average (range: 1 to 27 days) after the fracture. Stable condition meant that the patient began to be mobilized and could respond to questions.

We recruited the group without hip fractures from the patients in the Geriatrics Department of the same hospital. As soon as a patient with a hip fracture was enrolled in the study, we matched him or her with a non-fracture patient from the hospitalization list of the Geriatrics Department on the basis of age (mostly within 4 years, but up to 6 years in 13 patients older than 90 years for whom it was difficult to find controls) and sex. At the end of the study enrollment period, 215 patients were matched. The non-fracture patients were interviewed at an average of 17.5 days after the interview of the matched study patient.

### Mortality data

The date of death of the participants in both groups was obtained from the National Death Registry database, which includes the survival status of all citizens and is updated annually. We defined short-term mortality as death occurring less than 12 months after hip fracture and long-term mortality as death occurring 12 months or longer after hip fracture. To evaluate long-term mortality, we continued to follow the study participants who survived 12 months. The mean follow-up period was 46.1 months (range: 35 to 57 months). The mean survival time was 21 months (range: 3 days to 52 months) for mortality patients and 45.9 months (range: 35 to 57 months) for survival patients.

### Covariates

We included 55 covariates in our study (Table 1). All are important factors that might influence mortality status after hip fracture, according to previous studies and our own clinical experience. For consistent data gathering, trained study nurses used the same standardized questionnaire to interview patients in the hip fracture and the non-fracture groups. They collected information from patients or their proxy on demographics, self-reports of a physician’s diagnosis of underlying disease, current medications, health habits, nutritional supplements, cognitive function, physical functions, and living environment. Patients with a diagnosis of cancer, heart failure, angina pectoris, arrhythmia, liver disease, Parkinson's disease, or osteoarthritis were defined as participants with comorbidity. Bone mineral density (BMD) scans were arranged for all participants, and 341 of them agreed to the examination. All BMD data were measured by the dual-energy X-ray absorptiometry machine (Model: QDR4500A; Hologic, Waltham, MA, USA). T-scores were based on the total hip BMD (g/cm^2^) of the non-fractured side of the hip.

**Table 1:** Covariates representing the patient’s condition before hip fracture

**Type**	**Covariates**
Demography	**Age**, **sex**, **ethnicity**, **occupation**, **marital status**, **living arrangements**
	**Cancer**, **heart failure**, **angina pectoris**, **arrhythmia**, **liver disease**, **Parkinson's disease**, **osteoarthritis**
Medication	**Antihypertensive**, **cardiovascular medications**, **analgesics**, **anti-diabetes**, **psychotropics**, **gastrointestinal**, **other medications**, **polypharmacy (≥4 medications)**
Health habits	**Cigarette smoking**, **alcohol intake, betel nut chewing**, **leisure time physical activity**
Diet and nutrition	**Vegetarian diet**, **use of milk**, **coffee**, **tea**; use of nutritional supplements such as **calcium**, **multivitamin**, and **glucosamine**
Falls and fracture	**History of fall**, **history of fall-induced fracture**, **locations of fracture**, **place**, and **time** that fall happened
Living environment	**Building type**, **floor on which the participant lived**, **number of stairs in a flight**, **self-evaluation of stair height, stair lighting**, **outdoor lighting**, **green light duration**
Physical functions	**ADL**[19]: eating, bathing, dressing, toileting, getting in and out of bed
	**IADL**[20]: preparing a meal, shopping, using a telephone, taking medicine, light and heavy housework
	**Mobility tasks**[21]: bending, walking from room to room, walking up 10 steps, walking a quarter of a mile, grasping, lifting 10 pounds, raising arms over head, unlocking with a key
	**Hearing** and **visual ability**, **finger-nose-finger coordination**[22]
Cognitive functions	**MMSE**[23]
Anthropometric	**Body height**, **body weight**, **BMI**
Other	**BMD (T-score)**

ADL: activities of daily living; IADL: instrumental activities of daily living; MMSE: Mini Mental State Examination; BMI: body mass index; BMD: bone mineral density.

The 55 covariates were shown in bold type.

### Statistical analysis

We used the Kaplan-Meier survival curve to analyze the mortality rates of the fracture and non-fracture patients, and then compared them using the log rank test. To study how the covariates affect short- and long-term excess mortality, we used the Cox proportional hazards model to calculate hazard ratios (HR) of excess mortality and 95% confidence intervals (CIs) for the covariates. We defined death as the outcome event and survival time as the duration from hip fracture to the date of death. Patients’ data were censored if they were still alive at the end of the study. First, we used the model to test the unadjusted HRs of all 55 covariates for the entire follow-up period. Significant continuous covariates in the first model were converted to dichotomous variables using a cutoff value determined by the Youden index created by a receiver operating characteristic (ROC) curve [24].

The cutoff values of the significant continuous covariates were 84 years for age, 19 for Mini Mental State Examination (MMSE) score, −2.19 for T-score, and 20 for body mass index (BMI). We tested these covariates using graphical methods, all of which were compatible with the proportional hazards assumption. Univariate analysis using the Cox proportional hazards model was performed to test the unadjusted HRs of each covariate both for short-term and long-term excess mortality. The significant covariates generated in univariate analysis were then entered into the next model.

We performed multivariate analysis using forward stepwise Cox regression model to calculate the adjusted HRs. With *p* values set at 0.05 for entry and 0.1 for removal, the adjusted HRs were generated in this model. The PARP indicated that the rate of death in the entire population could be reduced if the risk factor for excess mortality were absent [25,26]. The PARPs were determined according to the adjusted HRs and calculated as P × [HR-1]/HR, where P is the exposed number of patients who died divided by the total number of patients who died. All analyses used the SPSS software (statistical software package for Windows version 19.0, IBM, Chicago, IL, USA) and the MedCalc software program (version 11.2, MedCalc, Mariakerke, Belgium).

## Results

Among the 432 patients enrolled in the study, 305 (70.6%) were women, and the mean age of all patients was 79.3 ± 7.5 years, with a range of 60 to 99 years. Patients with hip fracture had significantly lower BMI, T-score, and MMSE; worse activities of daily living (ADL) function, instrumental activities of daily living (IADL) function, coordination, and mobility function; less coffee drinking; and less participation in physical exercise than did their age- and sex-matched controls (Table 2). There were no significant differences in the proportions of having comorbidity and smoking.

**Table 2:** General characteristics of the study population

Variable*	**Overall (%)**	**Hip fractures (%)**	**Non-Hip fractures (%)**	***p *** **value**
Age (years)				Matched
≤ 84	329 (76.2%)	147 (67.7%)	182 (84.7%)	
> 84	103 (23.8%)	70 (32.3%)	33 (15.3%)	
Sex				Matched
Female	305 (70.6%)	156 (71.9%)	149 (69.3%)	
Male	127 (29.4%)	61 (28.1%)	66 (30.7%)	
BMI				
>20	350 (81.0%)	160 (73.7%)	190 (88.4%)	
≤20	82 (19.0%)	57 (26.3%)	25 (11.6%)	<0.001
T-score				
> −2.19	199 (46.1%)	49 (22.6%)	150 (69.8%)	
≤ −2.19	142 (32.9%)	95 (43.8%)	47 (21.9%)	<0.001
Missing	91 (21.1%)	73 (33.6%)	18 (8.4%)	
MMSE				
>19	270 (62.5%)	102 (47.0%)	168 (78.1%)	
≤ 19	162 (37.5%)	115 (53.0%)	47 (21.9%)	<0.001
ADL difficulty				
No	346 (80.1%)	157 (72.4%)	189 (87.9%)	
Yes	86 (19.9%)	60 (27.6%)	26 (12.1%)	<0.001
Coordination abnormality				
No	376 (87.0%)	175 (80.6%)	201 (93.5%)	
Yes	56 (13.0%)	42 (19.4%)	14 (6.5%)	<0.001
Weight-bearing exercise in past 2 weeks				
No	204 (47.2%)	118 (54.4%)	86 (40.0%)	
Yes	228 (52.8%)	99 (45.6%)	129 (60.0%)	0.003
Coffee drinking				
No	312 (72.2%)	176 (81.1%)	136 (63.3%)	
Yes	120 (27.8%)	41 (18.9%)	79 (36.7%)	<0.001
Comorbidity				
No	214 (49.5%)	99 (45.6%)	115 (53.5%)	
Yes	218 (50.5%)	118 (54.4%)	100 (46.5%)	0.124
Smoking				
No	325 (75.2%)	160 (73.7%)	165 (76.7%)	
Yes	107 (24.8%)	57 (26.3%)	50 (23.3%)	0.504

BMI: body mass index; MMSE: Mini Mental State Examination; ADL: activities of daily living.

*Only age, sex, the variables with significant difference between two groups and the significant variables affecting the excess mortality were listed.

**Figure 1:**
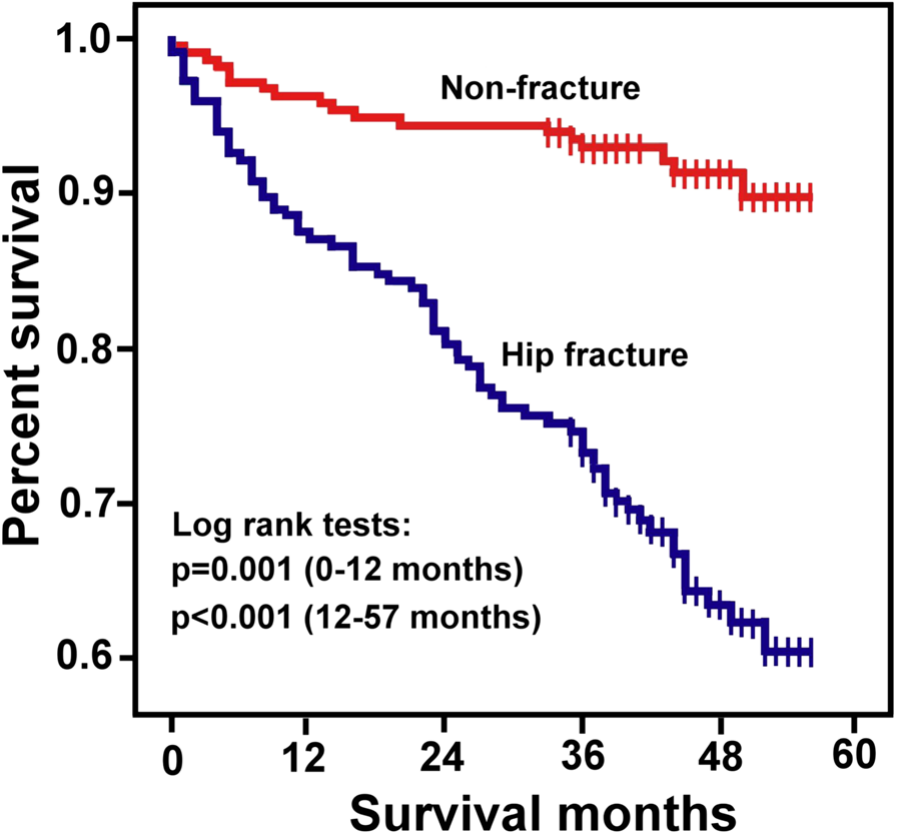
**Kaplan-Meier survival curve.** Five-year Kaplan-Meier estimates of cumulative probability of survival after hospital admission for hip fracture. Each vertical tick mark indicates a follow-up month in which patient censoring took place.

During the follow-up, there were 93 (21.5%) deaths for all the enrolled patients. Among them, 75 (34.6%) patients with hip fractures died versus 18 (8.4%) patients without fractures. The Kaplan-Meier survival curve of the hip fracture group declined after the first month of follow-up and significantly diverged from the curve of the non-fracture group (Figure 1). In the short-term mortality study, the overall mortality rate was 8.1%. In the hip fracture group, 12.4% of those patients died versus 3.7% in the non-fracture group (HR 3.5; 95% CI 1.6-7.6).

Of the five significant factors contributing to short-term mortality in univariate analyses, three covariates remained significant in the forward stepwise Cox regression model (Table 3): hip fracture (HR 2.4; 95% CI 1.1-5.4), presence of comorbidity (HR 2.3; 95% CI 1.1-4.7), and lower MMSE score (HR 2.3; 95% CI 1.1-4.8).

**Table 3:** Cox regression analysis showing hazard ratio for short-term mortality after hip fracture

**Variable**	**Survivals (%)**	**Deaths (%)**	**Unadjusted HR (CI)**	***p *** **value**	Adjusted HR^* ^**(CI)**	***p *** **value**	**PARP (CI)**
Hip fracture							
No	207 (96.3)	8 (3.7)	1.0		1.0		
Yes	190 (87.6)	27 (12.4)	3.4 (1.6-7.7)	0.002	2.4 (1.1-5.4)	0.037	44.7% (3.3-74.1)
Comorbidity							
No	203 (94.9)	11 (5.1)	1.0		1.0		
Yes	194 (89.0)	24 (11.0)	2.2 (1.1-4.5)	< 0.001	2.3 (1.1-4.7)	0.028	38.1% (4.5-65.0)
MMSE							
> 19	258 (95.6)	12 (4.4)	1.0		1.0		
≤ 19	139 (85.8)	23 (14.2)	3.3 (1.7-6.8)	< 0.001	2.3 (1.1-4.8)	0.022	34.3% (5.6-64.0)

HR: Hazard ratio; CI: 95% confidence intervals; PARP: population attributable risk proportion; MMSE: Mini Mental State Examination.

^*^Multivariate adjustment for the significant risk factors in univariate analysis; only significant variables in this model were listed. Lower body mass index and lower T-score were only significant in univariate analyses.

The 397 patients who survived through the first year were further evaluated for long-term mortality. The overall long-term mortality was 14.6%, with 25.2% of the patients in the hip fracture group dying versus 4.8% in the non-fracture group (HR 5.4; 95% CI 2.7-10.7).

Of the eleven significant factors contributing to long-term mortality, six covariates remained significant in the forward stepwise Cox regression model (Table 4): hip fracture (HR 2.7; 95% CI 1.3-5.6), lower T score (HR 3.3; 95% CI 1.4-7.8), lower BMI (HR 2.5; 95% CI 1.4-4.3), the presence of comorbidity (HR 2.1; 95% CI 1.9-3.6), ADL difficulty (HR 1.9; 95% CI 1.1-3.4), and smoking (HR 2.5; 95% CI 1.4-4.4).

**Table 4:** Cox regression analysis showing hazard ratios for long-term mortality after hip fracture

Variable^*^	**Survivals (%)**	**Deaths (%)**	**Unadjusted HR (CI)**	***p *** **value**	Adjusted HR^* ^**(CI)**	***p *** **value**	**PARP (CI)**
Hip fracture							
No	197 (95.2)	10 (4.8)	1.0		1.0		
Yes	142 (74.7)	48 (25.3)	5.4 (2.7-10.7)	< 0.001	2.7 (1.3-5.6)	0.007	48.0% (17.5-75.9)
BMI							
> 20	45 (64.3)	25 (35.7)	1.0		1.0		
≤ 20	294 (89.9)	33 (10.1)	4.0 (2.4-6.8)	< 0.001	2.5 (1.4-4.3)	0.002	42.8% (12.8-53.5)
T-score							
> −2.19	183 (95.3)	9 (4.7)	1.0		1.0		
≤ −2.19	93 (75.6)	30 (24.4)	6.1 (2.9-12.9)	<0.001	3.3 (1.4-7.8)	0.017	36.2% (11.7-56.2)
Missing	63 (76.8)	19 (23.2)	5.8 (2.6-12.8)		3.3 (1.3-7.4)		
Comorbidity							
No	184 (90.6)	19 (9.4)	1.0		1.0		
Yes	155 (79.9)	39 (20.1)	2.3 (1.3-4.0)	0.003	2.1 (1.9-3.6)	0.011	34.8% (8.6-57.4)
ADL difficulty							
No	288 (89.4)	34 (10.6)	1.00		1.0		
Yes	51 (68.0)	24 (32.0)	3.4 (2.0-5.7)	< 0.001	1.9 (1.1-3.4)	0.017	31.8% (3.1-37.9)
Smoking							
No	265 (87.5)	38 (12.5)	1.0		1.0		
Yes	74 (78.7)	20 (21.3)	1.7 (1–2.9)	0.051	2.5 (1.4-4.4)	0.001	19.2%
							(6.7-36.1)

HR: Hazard ratio; CI: 95% confidence intervals; PARP: population attributable risk proportion; BMI: body mass index; ADL: activities of daily living.

^*^Multivariate adjustment for all other significant risk factors in univariate analysis; only significant variables in this model were listed. Coordination abnormality, lower Mini Mental State Examination score, older age, vegetarian diet, and less participation in physical exercise were only significant in univariate analyses.

In PARP analyses, after adjusting for other significant risk factors, the risk factor contributing the most to excess mortality was hip fracture, with a PARP of 44.7% for short-term excess mortality and 48% for long-term excess mortality. The PARPs for other factors contributing to short-term excess mortality were 38.1% for comorbidity and 34.3% for lower MMSE. The PARPs for other factors contributing to long-term excess mortality were 42.8% for lower BMI, 36.2% for lower T-score, 34.8% for comorbidity, 31.8% for ADL difficulties, and 19.2% for smoking.

## Discussion

In this prospective, hospital-based cohort study for hip fractures among the elderly, survival analyses showed a higher short- and long-term excess mortality in the hip fracture patients than the sex- and age-matched non-fractured patients. After adjustment for other potential risk factors and calculation of PARP, hip fracture was still a significant risk factor and contributed the most to both short-term and long-term excess mortality.

Mortality rate as a direct and simple summary of mortality information can be a general indicator of the health status of a geographic area or population. In the present study, the short-term and long-term morality rates in the hip fracture population were 12.4% and 25.3%, respectively. Both rates were somewhat lower than almost all those previously reported, which might be up to 50% and 52%, respectively [8-12,16,27]. The difference might be explained by better care in a medical center like our hospital, in which the patients received high quality care given by experts from a wide range of medical specialities. It should be noted that the short-term mortality rate has also been reported to be as low as 9.2% [28], which may be attributable to long hospital stay [29]. Mortality rate alone, however, does not reflect how many excess deaths are attributable to hip fractures and cannot provide information on the relative importance of the injury.

The excess mortality rate is more meaningful clinically than the mortality rate alone. Quantification of additional deaths makes it possible to evaluate the relative influence of hip fracture or other risk factors on the mortality of patients. It can be expressed as an absolute effect or a relative effect. Absolute effect is the actual difference in the mortality risk between an exposed and an unexposed population. Relative effect is expressed by the hazard ratio of the mortality risk between an exposed and an unexposed population. The absolute effect provides straightforward information about excess mortality that is particularly useful when considering trade-offs between likely benefits and likely harms of an intervention [30]. Still, the clinical importance of the excess mortality may depend more on the relative effect than the absolute effect alone, especially when the mortality rate is low [31].

In the present study, for short-term excess mortality, the absolute effect was 8.7%, but the relative effect could be up to 3.4-fold. For long-term excess mortality, the absolute effect was 20.5%, but the relative effect was up to 5.4-fold. This relative effect provides more information on the importance of the hip fractures and has the additional advantage of allowing a direct comparison among different studies. In contrast to absolute effect, relative effect can also be adjusted by the inclusion of other competing risk factors through multivariate analysis [32].

Although studies have consistently reported significant short-term excess mortality after hip fractures [1,14-18,33-35], reports of long-term excess mortality have widely varied. Some studies that adjusted only for age and sex reported hip fracture was a significant risk factor for long-term excess mortality, but their conclusions may be biased because of the absence of adjustment for other potential risk factors [7,12,17,33]. Some other studies that did adjust for the potential risk factors found that hip fracture was statistically significant for long-term excess mortality [18,35-37], while others had an opposite finding. [13,14,27]. All of these studies used secondary data from the disease registry, hospital records, or data that were not originally collected for the purpose of studying excess mortality. Consequently, information on risk factors collected in these studies might not be comprehensive enough to allow adequate adjustment. Moreover, in the secondary data, the conditions of the patients recorded at the beginning of data collection might have changed by the time of the hip fracture and thus could affect the accuracy and reliability of the study results. To date, there has been a lack of primary data studying long-term excess mortality of hip fractures and our report helps fill that gap in knowledge.

Another issue related to assessment of long-term excess morality in patients with hip fracture has to do with the optimal selection of the control group. Most reported excess mortality studies compared hip fracture patients with a healthy general population. However, hip fracture patients have worse health conditions than general population. The problem with using these two groups for comparison is that the significance of the hip fracture might be confounded by comorbidities [14,38]. In our study, hip fracture patients were compared with hospitalized non-fracture patients recruited from the geriatric department. The percentage of patients with comorbidities was similar in these two groups (54.4% vs. 46.5%, *p* = 0.124). By ensuring that the study subjects were relatively homogeneous, we maximized the chance of detecting important etiological factors through multivariate analysis [39]. Having done that, our results confirmed the finding that hip fracture itself can cause a significant long-term excess mortality. The reasons for the association of high long-term excess mortality and hip fracture can be severe post-fractural complications such as pulmonary embolism, infection, or cardiovascular events and residual morbidities such as loss of mobility, chronic pain, and so forth [37].

Although interest in quantifying the impact of risk factors on the population is increasing among policy makers in public health, very few studies to date have reported using PARP to evaluate excess mortality after hip fractures. Tosteson et al. reported PARPs of hip fracture for excess mortality ranging from 0.5% to 6% [14]. In a study of osteoporotic fractures, Bliuc et al. reported PARP for excess mortality to be 18% for lower T-score and 10% for smoking [34]. In our study, after adjustment for a wide range of risk factors, hip fracture had the greatest impact on excess mortality with PARP of 44.7% for short-term and 48% for long-term mortality. The estimate of PARP of hip fractures in the present study was much higher than that in previous studies possibly because of oversampling of hip fracture patients. However, with an aging population and the increasing incidence of hip fractures, our finding of high PARP of fracture itself strongly supports the importance in our public health care system of hip fracture prevention in the elderly and adequate treatment when it occurs.

Our study has several strengths. First, we incorporated detailed objective information on as many as 55 covariates. To the best of our knowledge, this study is the first primary research project focusing on the specific issue of long-term excess mortality associated with hip fractures. Second, ours is also the first study using hospital-based controls with similar comorbid conditions to evaluate excess mortality of hip fractures. Third, this is the first study to report the PARP of each covariate for excess mortality. Fourth, the death data obtained for the present study were maintained by the government for administrative use and thus were very reliable. In some other studies, the survival status of the patients obtained by telephone calls or postcards might be incorrect because of no validation [7,16,37]. Finally, the included subjects in this study were representative of all the hip fracture patients admitted in our hospital. The patients’ refusal to join the study was based on their personal choice, and their baseline conditions were not significantly different from that of the study subjects.

Limitations of the present study should be noted. First, the study subjects were community-dwelling patients in one medical center, and so caution is necessary in generalizing the estimate of excess mortality to other populations. Second, because of the limited number of deaths, the effects of some risk factors in our study might not have been detected. The identification of fewer factors in short-term mortality might be caused by the small number of deaths in the first year. According to a power study for the proportional hazards model, the events per variable should not be less than 10 [40]. In our study, the number of short-term deaths in the first year was 35, and only three risk factors were identified. For long-term death, the number of deaths was 58, and six factors were identified. Third, low T-score, low BMI, ADL difficulty, and smoking had a significant impact on long-term excess mortality, but not on short-term mortality. This finding is somewhat different from previous studies [33,41-43], possibly because risk factors such as underlying health, immobility, etc. might be mutally correlated and replace one another in the regression models. Fourth, the lack of BMD data for 21.1% of our patients might reduce the accuracy of our risk estimation. Fifth, the excess mortality estimated in our study might be affected by patients’ recall bias in responding to the questionnaires. Sixth, although the age difference between the fracture and non-fracture groups might be up to 6 years in very old patients, this would not affect the study results significantly. Other possible biases might be also caused by imprecision of the measurements of the risk factors in both fractures and non-fractures. However, the misclassification might be non-differential and tend to result in underestimating the effect of risk factors. Last, in addition to the mortality risk factors covered in the present study, there are other potential risk factors that were not included. However, the present study focused on those factors that are reliable, easily accessed, and modifiable.

## Conclusions

The excess mortality after hip fracture may last longer than 57 months. Even after adjustment for comorbidities and other baseline conditions, hip fractures still can result in both short- and long-term excess mortality. With hip fracture having the highest impact on excess mortality, its adequate prevention and treatment should be targeted.

## Abbreviations

PARP: Population attributable risk proportion; BMD: Bone mineral density; HR: Hazard ratio; CI: Confidence interval; SPSS: Statistical product and service solutions statistical software; ROC curve: Receiver operating characteristic curve; MMSE: Mini mental state examination; BMI: Body mass index; ADL: Activities of daily living; IADL: Instrumental activities of daily living.

## Competing interests

The authors declare that they have no competing interests.

## Authors' contributions

L.W.H., G.-S.H., W.J.T., and J.L. drafted the manuscript. L.W.H. and J.L. designed the study. L.W.H. and J.L. analyzed the data. All authors revised the manuscript. All authors read and approved the final manuscript.

## Pre-publication history

The pre-publication history for this paper can be accessed here:

http://www.biomedcentral.com/1471-2474/15/151/prepub
